# Surgery in London; Osteotemy; Callendar’s and Lister’s Methods of Treating Wouuds

**Published:** 1879-05

**Authors:** C. T. Parkes


### Foreign Correspondence.

### ARTICLE X.

### SURGERY IN LONDON. — OSTEOTOMY. — CALLENDAR'S AND LISTER'S METHODS OF TREATING WOUNDS.

*Messrs. Editors :*—With only the scanty time afforded by a two weeks' opportunity to judge of matters and things, I am justified in making one rather positive assertion, and that is, that no country's schools have better, if as good, clinical advantages as do these in London. Better results could not be effected with the material at the clinics than are there obtained by the renowned gentlemen conducting them. Assuredly surgery is here on the topmost wave of success and advancement. He who passes by London in the fond delusion of meeting with something better beyond, has failed to entertain, or be entertained by, the angel in disguise. Earnest men are here—learned, skillful men—men of genius and worth ; and their works are full of the richness of ripe experience and and thoughtful maturity. Always at work, never idle, ever alive, grand results must be the fruit of their incessant activity. The cordial good feeling and courteous treatment shown to a stranger by all these busy, and I may say overworked men, is beyond expression pleasant and cheering.

So far, I have visited only three or four hospitals, among them, of course, the oldest of all, St. Bartholomew's, always noted as the school of sound surgical teaching and successful surgical practice. I don't know why I came here first ; perhaps it was because I had a personal fondness for it, owing to the fact that Thomas Vicary, one of the earliest and most renowned teachers in this school and hospital, published the first work on anatomy written in the English language. As one treads the stones of the court of this hoary old structure, wanders through its halls or wards, or sits in its amphitheater, the very air seems heavy with truths evolved by its men of genius, and the walls echo the words of wisdom and honest instruction which issued from the lips of some of the greatest men whose triumphs have adorned the temples of our profession. It must be that the men who, in succession, follow in the footsteps of these great teachers, are aided and encouraged by the memories of the departed, to renewed determination of maintaining the glorious inheritance left them.

The happiest good feeling is shown to exist among the gentlemen associated together here. This fact will impress the visitor immediately. No self assertion, no spirit of criticism, each seems to vie with the other in offering assistance and sympathy. There is no solemn funereal silence maintained through an operation, but opinions are freely given and asked for by all present. It seems certain that good only, results from this freedom of consultation and mutual support. I have seen it nowhere else in any such degree as here, and its introduction as a customary method of action in other places, would eradicate stiffness, adverse criticism and many other of the unpleasant surroundings during public operations, to say nothing of the benefits which would assuredly follow its adoption.

Another good thing here. On every Thursday afternoon all the surgeons connected with the hospital appear, and all cases requiring operation are brought publicly into the theater, and an open consultation held upon them, every surgeon expressing his opinion on each case, the majority deciding what shall be done. The students being present reap the advantage of seeing the manner each one has of studying his cases, and the course of reasoning which leads up to the final conclusion.

The newest study presented to me so far has been that accompanying the comparatively new operation called osteotomy, done for the relief of deformity, either congenital, or acquired as the result of injury or disease. I call it comparatively new, because I believe that the last claimant for the honor of having originated, put into practice, and established the perfect feasibility of the operation, dates his first case sometime in 1876. It is certainly a very young operation to have come so early into almost universal adoption. Everybody here divides and cuts off bone in every possible direction, and for every possible trouble, with little more fear, apparently, of resulting harm than would probably follow paring a man's nails.

Mr. Callender, of St. Bartholomew's, did the operation on a boy about 12 years old, over a week ago. The little fellow was badly knock-kneed. The operator made an oblique incision from within downwards and outwards, over the tibia about one and one-half inches below the joint, freely exposing the bone. He partially divided the bone with a narrow chisel and then forcibly fractured it. The fibula did not give way at once, as frequently occurs, so Mr. C. cut down upon the fibula and divided it with the chisel. Then as the leg was prevented from coming to the same line as the femur by the external lateral ligament of the knee-joint, that ligament was divided subcutaneously, and the members of the entire extremity, placed in the same line. A long external splint extending from below the foot well up upon the body, was then applied, and the padding and rollers so arranged as to allow of easy dressing without disturbing the limb. The wounds were closed with sutures and covered with lint soaked in carbolized oil, 1 part to 10 or 12. The oil to be constantly renewed so as to keep the wound fresh and soft, but the other dressing on no account to be disturbed. I saw the boy again a week after the operation, the same blood-stained dressings were on the knee as when I saw him put to bed, only the most external had been taken away. The carbolized oil had been repeatedly applied. Everything was sweet about the wound, there was no evidence of inflammation ; the patient was comfortable and free from any pain. No attempt whatever was made to do an antiseptic operation. Mr. Callender insists on, provides for, and secures absolute rest for his patients, after and during the treatment of any injury. His success is as good as any other surgeon's with similar cases and like surroundings, and he certainly attributes very much indeed of this good result to the imposition and maintenance of perfect *rest* of the injured part, and of the entire body. It is the surgeon's duty to look to its requirement, to provide the means of securing it, and to be resolutely determined, that it shall be maintained. One who follows his cases and sees his results, cannot fail to be impressed with the truth of these principles which he so ably and incessantly inculcates.

I saw a case done by Mr. Lister at King's College Hospital on a man 30 or 40 years of age; both limbs were operated upon by Mr. Lister with all the antiseptic precautions and treatment so earnestly advocated by that gentleman. In this case, only a separation of the internal condyle of both femora was effected. The incision through the soft parts called subcutaneous, was made just opposite to the point where the condyloid surface becomes continuous with the surface of the shaft of the bone. A narrow, slender chisel was introduced into this opening, and the condyle separated from its attachment by driving the chisel into the bone at different points, until the condyle was all but free; then it was forcibly broken loose by drawing the leg strongly inwards. The leg was then easily placed in line with the femur. The chisel was not allowed to enter the joint or even to touch the catilaginous coverings of the end of the bone. Both limbs were then fixed in plaster of Paris casings from hip to ankle, a fenestra being cut out opposite to the seat of the wound. The wound was dressed antiseptically every day for a day or two, and then once a week or as often as occasion required. Mr. Lister showed in this case, that underneath the blood clot which filled up and to a degree overlapped the wound in the soft parts, the process of cicatrization was progressing rapidly without any evidence whatever of inflammation or suppuration. He said this often occurred in wounds dressed according to his method. He remarked also, that blood-clots in wounds were organized, in so far that their constituents were replaced by connective tissue cells, and the débris absorbed. No one can more carefully and certainly provide for free drainage, than does Mr. Lister, in every wound that he makes. If there is any one detail of operative procedure to which he devotes more attention than drainage, it is close and accurate adjustment of the edges of wounds. Relief of tension is always provided for, by sutures introduced far away from the edges; they are always of wire, and held in place by oval shaped leaden plates, 2 Cm. wide and 4 Cm. long. The interrupted sutures are placed near to each other between these long and deep ones, for preventing tension. Catgut ligatures are used to control hæmorrhage. He showed several other cases of osteotomy, all doing well. From what has been seen thus far, the conviction is that anything like an ill result is the grand exception, after these operations on bone. Last Thursday, at the consultation at St. Bartholomew's, Mr. Callender presented a boy aged 6 or 7 years, who had met with a fracture of both bones of the forearm sometime previously. He had received no treatment from a surgeon, and union had taken place with great deformity, sufficient to interfere with and prevent rotation of the forearm. He proposed to cut down upon the bones, divide them with the chisel, place them in proper position, and treat as in an ordinary fracture. He preferred this to the old method of forcible fracture, because he in this way would know just what injury he was inflicting, and felt no concern but that surely a good result would follow the operation. All these cases are practically compound fractures of bone, and to hear these men talk so unreservedly, and with so much evident satisfaction and confidence, about the result, after surgically producing the injury, is one of the many revelations which have been presented to my vision; and seemingly the bolder the surgical proceeding, the better and more satisfactory the result.

Last Wednesday I saw Mr. Lister make an amputation at the hip-joint for osteo-sarcoma of the lower end of the femur. It was of immense size, and of very rapid development; the history included only a period of seven months. The patient was a woman of about 40 years of age. Mr. L. made an ordinary amputation of the thigh inside of the upper third, as the first step of the operation. After carefully controlling all bleeding vessels in this section, he removed the remainder of the femur by means of a long incision down the outer aspect of the stump, which enabled him to dissect out and disarticulate the remnant of the bone. He remarked that he thought this the best method to avoid excessive loss of blood and shock. The parts being extremely vascular, over forty ligatures were required to stop bleeding. The wound looked almost as " wide as a church door, and as deep as a well," as it lay gaping on the table; a fearful cut indeed. All the details of antiseptic dressing were carried out as they can only be by the originator of the system. If you were to ask me what I think of antiseptic dressings so far, I should answer that I am still hesitating between opinions. I will tell you why, by giving you an account of the result of two quite similar operations, done, one by a non-believer, and the other by Mr. Lister. The operation in both cases was for very extensive infiltrating carcinoma of the female breast, complicated with enlarged glands in the axilla, the removal of which required very extensive incisions and dissections. Mr. Callender did his operation without any reference whatever to antiseptic dressing, provided carefully for drainage by means of tubing, and brought the edges of the wound into close approximation. The dressing consisted of several double folds of lint soaked in carbolized oil, and covered with oil silk all held in position by adhesion strapping, and the arm carefully fixed to the patient's side—everything done to provide for perfect rest. I saw the patient the next day, and was present when the wound was dressed. The patient was absolutely free from all pain during the whole proceedure, in fact the only thing required, was to cut the plaster strips as they passed beyond the lint folds, and the dressing was lifted off without any trouble whatever. The oil of course had prevented drying, and been a soothing application. This oil is applied by means of long brushes, and nothing harsh of any kind is allowed to touch the wound or patient. By this means the patient's confidence is positively secured, and any fear of pain on his part done away with. No one can deny that such a result is of incalculable advantage to the patient after any operation, and certainly aids the recovery. The dressings are easily secured again by short strips of plaster carried over the old plaster left in position. These first plaster strips are left on until the cure is complete, never torn away while the wound is fresh and tender, a process which always brings agony to the patient. After 24 hours this case was in every way entirely comfortable. Very slight elevation of temperature, less than 38° ; during the entire next week this gradually and steadily sank to normal. I saw the patient at the end of the week, the wound all healed, sutures all removed—no pus—not anything but comfort and cheerfulness on the part of the patient. No result could be better than this, and yet how simple the treatment, how plain and easily fulfilled the requirements! Mr. Callender says these results are common with him, and with his statement to support me, I assert freely, that this is the best and only method of treating wounds.

Mr. Lister did his operation of exactly the same kind and nature, with all his great skill and care. Everything worked to a charm, there was no abscence of spray, everything used was rendered thoroughly aseptic. Mr. L. used similar, if not greater care, than we have noticed in the other case, in providing for drainage, and in the approximation of the wound. The patient's chest and corresponding arm were swathed in the gauze dressing, which was secured in position first by gauze bandage, and then finally by a few turns of the elastic roller, which prevents it from becoming displaced by any movements of the patient. I saw the dressing re-applied a few days after. There was no putrefaction. The wound looked perfectly healthy, but the patient was not as comfortable as in the other case. The change of the dressing was immensely more difficult for the surgeon, and as certainly more painful for the patient, because far more movement on her part was necessitated in the removal and re-application of the gauze, even with so skilled a director as Mr. Lister to superintend. Mr. Lister says pyæmia has passed away, erysipelas is a thing of olden times; ary cavity of the body, joint or otherwise, can be freely opened and examined with impunity and perfect confidence, under the protecting wing of antiseptic dressing. With this statement to support, comes the second assertion — every surgeon ought to master the principles of this most masterly system of safety in operation, and after injuries.

Many surgeons here do not use the antiseptic method of Mr. Lister at all; many in a slipshod manner; none like Mr. L. himself.

C. T. PARKES.

